# Alpha-Fetoprotein and Novel Tumor Biomarkers as Predictors of Hepatocellular Carcinoma Recurrence after Surgery: A Brilliant Star Raises Again

**DOI:** 10.1155/2012/893103

**Published:** 2012-06-27

**Authors:** Quirino Lai, Fabio Melandro, Rafael S. Pinheiro, Andrea Donfrancesco, Bashir A. Fadel, Giovanni B. Levi Sandri, Massimo Rossi, Pasquale B. Berloco, Fabrizio M. Frattaroli

**Affiliations:** ^1^Department of General Surgery and Organ Transplantation, Sapienza University of Rome, Umberto I Policlinic of Rome, Viale del Policlinico 155 00161, Rome, Italy; ^2^Department of Liver Transplantation, University of São Paulo, Av Dr Eneas de Carvalho Aguiar 255, 05403-010 São Paulo, Brazil; ^3^Department of Surgery, Arzignano, Hospital, ULSS5 Ovest Vicentino, Via Kennedy 2 36071, Arzignano, Italy; ^4^General Surgery Department, Assiut University Hospital, Assiut 71515, Egypt

## Abstract

Alpha-fetoprotein (AFP), des-**γ**-carboxy prothrombin (DCP), and lens culinaris agglutinin-reactive fraction of AFP (AFP-L3) have been developed with the intent to detect hepatocellular carcinoma (HCC) and for the surveillance of at-risk patients. However, at present, none of these tests can be recommended to survey cirrhotic patients at risk for HCC development because of their suboptimal ability for routine clinical practice in HCC diagnosis. Starting from these considerations, these markers have been therefore routinely and successfully used as predictors of survival and HCC recurrence in patients treated with curative intent. All these markers have been largely used as predictors in patients treated with hepatic resection or locoregional therapies, mainly in Eastern countries. In recent studies, AFP has been proposed as predictor of recurrence after liver transplantation and as selector of patients in the waiting list. Use of AFP modification during the waiting list for LT is still under investigation, potentially representing a very interesting tool for patient selection. The development of a new predictive model combining radiological and biological features based on biological markers is strongly required. New genetic markers are continuously discovered, but they are not already fully available in the clinical practice.

## 1. Alpha-Fetoprotein: A Historical Background

*Alpha-fetoprotein* (AFP) has been considered for a long time the ideal serological marker for detecting hepatocellular carcinoma (HCC).

It is well known that persistently elevated AFP levels are related to the presence of HCC and that its determination can be helpful for a better definition of at-risk patients (i.e., patients with a history of cirrhosis) [1]; hence, AFP is the most widely tested biomarker in HCC. 

Historically, AFP has been mainly tested in the diagnostic mode rather than for HCC surveillance after any type of tumor treatment; consequently, AFP has been introduced as a variable in the flowcharts used for HCC diagnosis in both Europe and the USA [2, 3].

However, when combined with ultrasounds (US), AFP levels have shown to be able to only provide additional detection in 6–8% of not previously identified cases [4]. 

Several aspects are at the basis of the suboptimal performance of AFP as a serological test for HCC diagnosis.

Firstly, HBV or HCV infection and exacerbation of underlying liver disease determine fluctuating levels of AFP in patients with cirrhosis [5, 6]. 

Secondly, only a small proportion of early-stage HCCs (10–20%) present with abnormal AFP serum levels [7]. 

When used as a diagnostic test, AFP value of 20 ng/mL shows good sensitivity but low specificity, whilst the higher cut-off value of 200 ng/mL presents a high specificity but a sensitivity dropping to 22% [8]. 

## 2. The Demise of a Brilliant Star: AFP Is Removed from Diagnostic Flowcharts

Starting from these considerations, AFP has not been considered anymore a valid test to recommend for the survey of patients at risk of developing HCC. 

In a recent publication, Forner et al. have poetically defined AFP discontinuation as marker for HCC diagnosis as a “demise of a brilliant star” [9].

**Figure 1:**
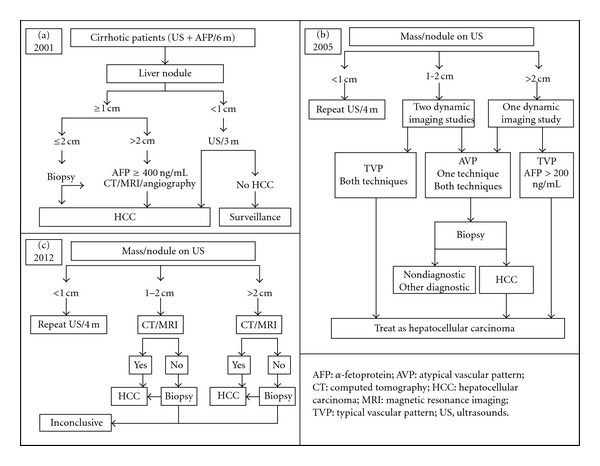
Development of different European and US flow charts proposed in the last decade for the diagnosis of HCC. AFP has progressively disappeared from the variables used for the diagnosis. Flow-chart (a): from Bruix et al. EASL guidelines 2001 [2], with modifications; flow chart (b): from Bruix et al. AASLD guidelines 2005 [3], with modifications; flow-chart (c): from EASL-EORTC guidelines 2012 [4], with modifications.

Looking at the flowcharts used for HCC diagnosis, the most recent ones consider only radiological exams, eventually combining biopsy in the diagnostic process in the presence of tumors with uncertain behavior or very small dimensions (Figure 1) [4]. 

## 3. Introduction of New Biomarkers in HCC Diagnosis

Apart from AFP, several novel markers have been developed in the last years with the intent to improve the diagnostic power and to better detect HCC in the population of cirrhotic patients.

Among them, *des-*γ*-carboxy prothrombin* (DCP) and *lens culinaris agglutinin-reactive fraction of AFP* (AFP-L3) have been more largely investigated, in both Western and Eastern experiences [10, 11].

(1) DCP, also called prothrombin induced by vitamin K absence (PIVKA), is an abnormal prothrombin molecule generated as a result of an acquired defect in the posttranslational carboxylation of the prothrombin precursor in malignant cells: this decarboxylated prothrombin is also produced in the presence of vitamin K deficiency [12].

The exact cause of DCP production in HCC has not been completely understood yet. Several possible explanations have been proposed: (a) activity of carboxylase enzymes declines into the tumor tissue; (b) a splice variant of carboxylase is present in DCP-producing HCC cells; (c) availability of vitamin K declines in the tumor as the result of an abnormal vitamin K metabolism [13].

In recent studies, vitamin K administration has been correlated with a dose-dependent reduction of DCP production [14, 15], suggesting that vitamin K may even present a biological effect against the tumor [15].

Clinical usefulness of DCP in HCC detection has been recently tested, showing major sensitivity and specificity respect to AFP [16, 17].

(2) AFP-L3 is an isoform of AFP and it is reported as the percentage of AFP-L3 over the total AFP level. Previous studies have identified 10% as the cut-off for the presence of HCC [18]. 

Many studies have investigated the role of AFP-L3, alone or in combination with AFP and DCP, as a marker for the surveillance of patients at risk for HCC [18–22].

Use of AFP-L3, despite its major diffusion in Eastern countries, has recently improved also in Western centres [19–22].

Contrasting results have been obtained comparing AFP-L3 with the other biomarkers: in a recent study, DCP has showed a higher sensitivity (87% versus 56%) and a superior ability in detecting patients without HCC [18], whilst in a US large phase 2 biomarker case-control study, AFP has been the most sensitive marker [19].

A prospective US study has shown a correlation between portal vein invasion and AFP-L3%, whilst DCP has been significantly associated with HCC metastasis [22].

However, despite the great interest related to the development of new biomarkers, DCP and AFP-L3 seem not to present markedly improved abilities in detecting HCC respect to AFP, mainly in the presence of early tumoral pathology. 

Both DCP and AFP-L3 fraction levels have been associated with portal vein invasion and advanced tumoral stage, a fact that prevents the usage of these markers for early detection [23, 24].

Consequently, the new serum markers, used alone or in combination, have not offered any substantial advantage with respect to AFP: at present, none of tests used for HCC detection can be recommended to survey cirrhotic patients at risk for HCC development [4].

## 4. The Star Rises Again: AFP as Predictor of Survival and Tumor Recurrence

In very recent years, AFP has been proposed as a predictor of patient survival and tumor recurrence after surgery, locoregional therapies, and systemic chemotherapy [25–29].

This new role derives from the strong correlation detected between AFP values, tumor dimensions, and microvascular invasion, all well-known predictors of HCC recurrence [30]. 

In fact, AFP represents a surrogate of tumoral activity and vascular invasiveness: AFP-mRNA dosage, used as a marker of HCC cell dissemination into the circulation, represents a further confirmation of this correlation [31, 32].

Many studies have investigated the best AFP measurement to consider for patient selection: in a recent large experience performed on 6817 US patients listed for liver transplantation (LT) with a diagnosis of HCC, last AFP before surgery has resulted as one of the strongest variables for patient selection before LT [25]. Similar results have been observed in a study from Italy, in which AFP before LT has been the unique independent risk factor for HCC recurrence [26]. 

Apart from the moment of AFP determination, a great debate also exists on the best AFP threshold value to be used. Several cut-offs have been proposed (210, 400 and 1000 ng/mL) [26, 33, 34], but none of them has already obtained a definitive international validation.

In two studies from France and Canada, the velocity of AFP increase has been suggested as the best predictor of HCC recurrence after LT. However, also this new parameter and its proposed cut-off value (15 or 50 ng/mL/month) need to be validated [35, 36]. 

## 5. Other Biomarkers as Predictors of Survival and Tumor Recurrence

In the last years, AFP, DCP, and AFP-L3 have been largely investigated with the intent to predict the risk of HCC recurrence after hepatic resection.

A long list of Eastern experiences confirmed the predictive role played by these markers in the preoperative time. A very recent study from Japan has observed that their triple positivity detected before hepatectomy was related to postoperative poor prognosis [11]. A similar analysis performed on 416 patients showed that AFP >100 ng/mL and AFP-L3 >15% before radiofrequency ablation were significant predictors for the risk of HCC recurrence [37]. 

The role of doubling time of preoperative AFP and DCP has been also investigated as useful tool for the prediction of early postoperative recurrence [38].

Two Korean studies performed on 126 and 245 resected patients showed that preoperative DCP was superior to AFP in predicting recurrence [39, 40]. In fact, recent researches have demonstrated that DCP stimulates human vascular endothelial cell growth and tumor migration, in this way explaining its important role as predictor of survival and recurrence [41]. As a confirmation, DCP was more specific for the detection of vascular invasion, whilst AFP-L3 was related to progression from moderately differentiated to poorly differentiated HCC [42].

Despite the large number of experiences reported, it is clear that not all the studies are in agreement among them: larger and possibly multicentre studies are needed with the intent to test the pre-operative role of these markers on a large cohort of patients, in this way avoiding the possible biases of patient selection.

Apart from their pre-operative detection, postoperative markers also showed a strong connection with HCC recurrence, even presenting in some cases a direct correlation between their value at recurrence and the site of recurrence [43].

Nanashima et al. observed that normalization of DCP after hepatectomy was significantly associated with good patient survival, in this way reflecting the efficacy of the treatment [44].

Postoperative DCP values appeared more closely associated with indices of tumor invasiveness like tumor large dimension, vascular invasion, intrahepatic metastases, and a lower grade of tumor cell differentiation [45]. One study performed on 124 patients treated with termoablation showed that posttreatment AFP-L3 resulted in the most reliable tumor marker for estimating overall survival and disease-free survival respect to AFP and DCP [46].

Despite the good results observed in all the reported studies, a recent report from the University of Nagasaki performed on 470 patients treated for HCC showed conflicting results, concluding that high AFP or DCP levels did not sufficiently reflect curative efficacy of treatment: hence, the authors affirmed AFP and DCP were poor predictors of prognosis in HCC patients [47].

Consequently, also for postoperative markers values, we can obtain the same considerations done for the pre-operative ones. A study performed on a large population is needed to better investigate the effective role of postoperative markers modification as predictors of recurrence. 

Apart from this, it is very interesting to underline that all the reported studies come from Asia, showing that the use of DCP and AFP-L3 represents a controversial point between Western and Eastern countries in the clinical management of HCC. In fact, despite the optimal results showed by some Eastern experiences, systematic application of these markers looks to be unlikely in European and US clinical practice. Possible explanations for this disagreement may be the poor availability of these new markers in Western countries, or the belief that Asian tumors are biologically different from Western ones and, consequently, with different biological proteins expression. 

It is opinion of the authors that a large evaluation of these markers also in the Western scenario is strongly required, with the intent to confirm their power for HCC recurrence prediction. 

## 6. Use of AFP and Other Markers in HCC Prognostic Staging Systems

In recent years, different staging systems have been proposed with the intent to better stratify HCC patients. In Japan, biomarkers have been integrated into some of them as selection variables.

For example, the proposed “biomarker combined Japan Integrated Staging (bm-JIS),” proposed in 2008, was invented with the intent to improve the stratification ability of the already existed JIS, in this way adding to the previous prognostic model the possibility of also estimating the HCC malignant grade. A total of 1.924 HCC patients were included in this study, and the three tumor markers AFP, AFP-L3, and DCP, were used. The stratification value of the bm-JIS score was superior to the JIS score, as observed by the higher likelihood ratio test obtained for the first model after Cox regression analysis [48].

Another score, called BALAD score, was developed in 2006. This scoring system was exclusively based on 5 serum markers: bilirubin, albumin, AFP-L3, AFP, and DCP. The system was validated in 2600 HCC patients from 5 institutions. The discriminative ability of the model resulted was excellent, adding the great advantage of being easy to perform [49].

Also in these cases, despite the large populations analyzed and the excellent ability in cohort stratification, the proposed scores need to be confirmed out of Japan, possibly in a Western experience, with the intent to confirm their effective pertinence. 

## 7. Use of AFP and Other Markers for the Selection of Liver Transplant Candidates

Use of AFP for the selection of LT candidates has recently observed a progressive increase. In a recent analysis performed on national data from the USA, transplanted HCC patients with moderately or highly elevated AFP levels presented poor results after LT. Moreover, patients with similar sized tumors but elevated AFP levels still had worse survival, showing that AFP seems to be a better surrogate for bad biology than size [50, 51].

In a very recent study from Europe, it has been clearly reported that *“use of changes in serum levels of biomarkers for assessment of response (i.e., AFP levels) is under investigation”* [4]; similarly, the US national conference for liver allocation in HCC patients has established that *“allocation points will be based on a candidate's calculated MELD score plus the following factors. *

- *AFP <500 ng/mL.*
- *Tumor size within the Milan Criteria (MC).*
- *Time within the MC (this includes patients down-staged to within the MC).*
- *No points will be added if the AFP level is greater than 500 ng/mL.” [34].*

As previously reported, studies from Italy, Switzerland, the USA, Canada, and France have been recently published, all of them underlying the role of AFP as predictor of survival and recurrence during the waiting time [25, 26, 33, 35, 36].

Combination of radiology and biology for the selection of LT candidates has been strongly proposed, in this way investigating not only the morphological features of the tumor, but also its biological aggressiveness (Table 1).

A study from North America has proposed the combination of total tumor volume (TTV) <115 cm^3^ and AFP <400 ng/mL for the selection of HCC patients, showing that patients exceeding these cut-offs presented very poor post-LT results (below 50% at 3 years) [33].

In another study from the Inter-University Consortium of Rome, 158 HCC patients were stratified according to the total tumor diameter (TTD) >8 cm and AFP >400 ng/mL. At multivariate analysis, both these variables were the unique independent risk factors for recurrence, presenting the AFP value >400 ng/mL an 8-fold increased risk for developing post-LT HCC recurrence. Radiological-biological combination consented to obtain a better selection of candidates for LT without worsening patient survival and recurrence rates, this approach allowing for an increase in the number of potentially transplantable patients [67].

Apart from AFP, also the other markers were used in LT: a study from Japan performed on 456 patients who underwent LT showed that the elevation of AFP-L3 (*P* = 0.0171) and DCP (*P* = 0.0004) significantly affected decreased survival rate and that DCP elevation had the strongest effect on patient survival [68].

In a study from Kyoto performed on 144 patients who underwent living donor LT, multivariate analysis revealed that tumor size >5 cm, > or = 11 nodules, and DCP >400 mAU/mL were significant independent risk factors for recurrence. The so-called Kyoto criteria were then developed according to these variables, consenting to obtain additional information regarding histological features and thus greatly improving patient selection criteria [69].

The recent progression in the number of studies focalized on the role of biological markers as selectors of patients waiting for LT strongly suggests that morphological aspects alone (i.e., tumor number and dimensions) are not fully able to select patients. Large national studies (mainly from North America) have been already performed, but an international validation of these new criteria is still needed, being nowadays MC (exclusively based on morphological aspects) the unique real criteria clearly recognized worldwide. 

## 8. The Future

Until now, an HCC molecular classification does not exist. In fact, HCC represents a complex and heterogeneous tumor with several genomic alterations. Many studies reported several aberrant activations of signaling cascades such as vascular endothelial and epidermal growth factor receptors (VEGFR and EGFR), Ras/extracellular signal-regulated kinase, phosphoinositol 3-kinase/mammalian target of rapamycin (mTOR), hepatocyte growth factor/mesenchymal-epithelial transition factor, Wnt, Hedgehog, and apoptotic signaling [70].

The recent introduction in the clinical practice of mTOR inhibitors and, mainly, sorafenib, as molecular therapies against HCC, has focalized on the attention on new plasma biological markers [71, 72].

However, an exponential increase in the number of new discovered proteins involved in tumor differentiation and aggressiveness has been observed in the very recent last years, further underlining the great complexity of HCC genomics and the difficulties existent in this area of research. 

In a recent analysis from the Sorafenib HCC Assessment Randomized Protocol (SHARP) trial, ten plasma biomarkers were tested in 491 patients [73]: two angiogenesis biomarkers (Ang2 and VEGF) were independent predictors of survival in patients with advanced HCC.

VEGF and its receptor (VEGF-R) seem to be extremely important in tumor aggressiveness, because of their angiogenetic role: VEGF represents the target molecule of sorafenib, the most important anti-HCC drug developed in the last years. 

Consequently, several studies have underlined the critical role of VEGF. In a study from Italy, a direct correlation between high AFP values and VEGF protein expression was reported [74]. Similarly, AFP mRNA and VEGFR-1 mRNA from the bone marrow and peripheral blood of 114 Japanese patients treated with primary curative hepatectomy showed a strong correlation of their concentration with the risk of early recurrence after surgical treatment [75].

The direct research of VEGFR expression in cell lines and specimens represents another attractive approach for investigating its role. High expression of VEGFR-1 was detected in 4 HCC cell lines and in the samples of 95 HCC patients treated with curative resection, resulting in an independent prognostic factor for recurrence and overall survival [76].

Besides the role of the new biomarkers evaluable from plasma or directly from the tumor tissue, global gene expression profiling may represent the most appropriate technology to unravel the pathogenesis of HCC and explore its heterogeneous origin [77]. In fact, it is very well known, as reported in a recent review by Marsh and Schmidt [78], that “In the field of surgical oncology, tumor biology is king, patient selection is queen, and technical maneuvers are the prince and princess who try, but usually fail, to usurp the throne.”

Unfortunately, although these investigations represent a promising progress in the field of prognostic prediction for HCC, their immediate application in the clinical practice is hindered by the great inhomogeneity existent among HCCs: in fact, a sort of “Babel” of different genetic subgroups exist according to etiological factors, tumor stages, recurrence and survival [79].

Recently, a real biobanking protocol has been established, with the intent to investigate whole genome sequencing, exome sequencing, gene-specific analysis, gene expression, and epigenetic analysis of HCC patients [80].

In the last decade, preliminary studies have been performed in both Western and Eastern centers. 

In 2003, Marsh et al. [81] proposed the use of a panel of tumor suppressor gene markers of allelic loss to better investigate the risk of tumor recurrence after LT. Loss of heterozygosity of specific genes of interest (APC, CDKN2A, DCC, MET, MYC1, OGG1, p34, p53, PTEN) was investigated in 103 different HCC patients. An index based on the fractional allelic imbalance (FAI) rate was constructed, showing a strongly accurate prediction for tumor recurrence.

FAI index was successively tested on 183 HCC patients who underwent LT [82], resulting the strongest predictor of post-LT recurrence, followed by vascular invasion and morphological aspects (tumor number and bilobar involvement).

Another study from the USA observed a gene signature based on 406 different genes able to discriminate two populations of good and poor survivals among HBV patients [83]. The same group, more recently, reported an additional hepatoblastoma-like HCC subgroup with very poor prognosis [84]. 

Other studies, using different genes, reported similar abilities in predicting patient and disease-free survivals and excellent accuracies [85–87]: for example, a Japanese study performed on 60 patients and based on a 12-gene signature identified through high-density oligonucleotide microarrays (>6000 genes) reported an accuracy above 90% [86]. 

A recent analysis performed on 214 resected patients, defined a gene-expression signature associated with the prediction of vascular invasion (accuracy: 69%) [88].

The recent discovery of RNA interference has further revolutionized the “loss of function genetics”: miRNA-based delivery strategies have shown antitumoral activity in HCC animal models, and dysregulation of miRNA has also proved high prognostic predictive value in human samples [89]. The use of reversible miRNA against P53 tumor suppressor has recently enabled the identification of P53 loss as a major requirement for the maintenance of murine liver carcinoma. Similar miRNA-based approaches were recently used to functionally validate DLC-1 as a tumor suppressor included in 8p, a chromosomal region that is found deleted in up to 50% of human HCC. 

The advent of further novel technologies, such as deep sequencing and integrative genomic analysis, and the consolidation of sophisticated animal models (mosaic models, transposons) will represent the beginning of a new era in cancer gene discovery. 

However, despite the surprising results reported using genomics and new target proteins involved in HCC pathways, a long time is still required before their routine use in clinical and surgical practice: the experience with sorafenib is still at the beginning, and new drugs will be developed in the next future. Larger validation of genetic aspects on international cohorts and common genetic markers between Western and Eastern patients are still required.

## 9. Conclusions

Biomarkers routinely used for HCC detection are suboptimal tests for routine clinical practice in HCC diagnosis. New and more accurate biomarkers for early HCC detection need to be already developed. On the contrary, these markers are routinely and successfully used as predictors of survival and HCC recurrence in patients treated with curative intent, mainly in Eastern countries. On these bases, the development of a predictive model combining radiological and biological features is strongly suggested. Use of AFP modification during the waiting list for LT is still under investigation. New genetic markers are continuously discovered, but their use is not already routinely available in the clinical practice.

## Figures and Tables

**Table 1:** Various proposed criteria for the selection of HCC patients waiting for liver transplantation.

Author (year), abbreviation	Criteria
Only radiological criteria	

Mazzaferro [52], Milan	1 HCC ≤5 cm or ≤3 HCC ≤3 cm
Yao [53], UCSF	1 HCC ≤6.5 cm or ≤3 HCC ≤4.5 cm with TTD ≤8 cm
Herrero [54], CUN	1 HCC ≤6 cm or ≤3 HCC ≤5 cm
Onaca [55], Dallas	1 HCC ≤6 cm or ≤4 HCC ≤5 cm
Sugawara [56], Tokyo	≤5 HCC ≤5 cm
Lee [57], Asan	≤6 HCC ≤5 cm
Silva [58], Valencia	≤3 HCC ≤5 cm with TTD ≤10 cm
Toso [59], TTV	TTV ≤115 cm^3^
Mazzaferro [60], Up-to-seven	Number + maximum size of HCC = 7
Fan [61], Shanghai	1 HCC ≤9 cm or ≤3 HCC ≤5 cm with TTD ≤9 cm

Criteria needing preoperative biopsy	

Cillo [62], Padua	Tumor grading I or II
Zheng [63], Hangzhou	TTD ≤8 cm or HCC grading I or II and AFP ≤400 ng/mL

Combined radiological and biological criteria	

Kwon [64], Seoul	HCC ≤5 cm (no number restriction) and AFP ≤400 ng/mL
Takada , Ito [65, 66], Kyoto	≤10 HCC ≤5 cm and DCP ≤400 mAU/mL
Toso et al. [33], TTV/AFP	TTV ≤115 cm^3^ and AFP ≤400 ng/mL
Lai et al. [67], TTD/AFP	TTD ≤8 cm and AFP ≤400 ng/mL

HCC: hepatocellular carcinoma; TTD: total tumor diameter; TTV: total tumor volume; AFP: alpha foetoprotein; DCP: des-*γ*-carboxy prothrombin.
